# Whose rights are being violated when receiving HIV and sexual and reproductive health services in Nigeria?

**DOI:** 10.1186/s12913-022-08624-9

**Published:** 2022-11-29

**Authors:** Morenike Oluwatoyin Folayan, Erva-Jean Stevens-Murphy, Ikenna Nwakamma, Joanne Lusher, Ibidunni Olapeju Oloniniyi

**Affiliations:** 1 Department of Child Dental Health, Obafemi Awolowo University, Ile-Ife, Nigeria; 2 Nigeria Institute of Medical Research, Yaba, Lagos State Nigeria; 3 Community Oral Health Department, Tehran University of Medical Sciences, Tehran, Iran; 4 Faculty of Health Sciences, University of Zaragoza, Zaragoza, Spain; 5 UNAIDS, UNAIDS, Nigeria; 6 Coalition of Civil Society Networks on HIV and AIDS, Nigeria, Nigeria; 7 Regent’s University, London, UK; 8 Department of Mental Health, Obafemi Awolowo University, Ile-Ife, Nigeria

**Keywords:** Rights violation, Patient’s rights violation, Transgender individuals, Sexual minority individuals, Living with HIV, Sexual and reproductive health services, Nigeria

## Abstract

**Background:**

In Nigeria, vulnerability status may increase the risk for the violation of human rights while receiving health care. The present study determined the proportion and profile of people who reported rights violation while accessing HIV and sexual and reproductive health (SRH) services.

**Methods:**

This was a cross sectional study with data collected between February and March 2021. The dependent variables were patients’ rights to autonomy: right to privacy and confidentiality of medical records; right to be treated with respect, regardless of gender, race, religion, ethnicity, allegations of crime, disability or economic circumstances; right to decline or consent to participation in medical research, experimental procedures or clinical trials; right to quality care in accordance to prevailing standards; and right to complain and express dissatisfaction regarding services received. The independent variables were sexual identity (heterosexual/straight, gay, lesbian, bisexual, queer), HIV status (positive, negative, do not know), living with disability (yes/no), and access point to HIV services (public or donor funded/private). Five multivariate regression models were developed to determine associations between the dependent and independent variable after adjusting for age, education level (no formal education, primary, secondary, tertiary), sex (male, female, intersex), marital status (single, married, separated/divorced, cohabiting) and gender identity (male, female, transgender).

**Results:**

Complete data from 2119 study participants were analysed. Transgender individuals had significantly higher odds of experiencing violation of their rights to privacy and confidentiality of medical records (AOR:1.70), right to be treated with respect (AOR:1.71), right to complain and express dissatisfaction regarding services received (AOR:1.57) and right to decline consent to participate in medical research, experimental research, experimental procedures or clinical trials (AOR:1.81) compared to individuals who were males.

**Conclusion:**

A high proportion of recipients of HIV and SRH services in Nigeria reported rights violations. Transgender individuals appear to have higher risk and those in spousal relationships have lower risk for rights violations. Studies are needed to learn how to improve rights-based HIV and SRH service delivery in Nigeria especially for transgender individuals.

## Introduction

Patients have a right to the respect of their dignity, confidentiality and consent to treatment when receiving health care services [1]. It is an important and discrete aspect of the right to health that conflates the concepts of consumer rights with civil and political rights, patient safety and bioethics [2]. The application of human rights principles to the context of patient care has grown in many parts of the world in response to the concerns about abuse when clients receive care [2].

Nigeria is one of several countries where patients report on the violation of their rights [3–6]. Such violations include, among others, violation of their right to privacy [7] and confidentiality of medical records [8]; right to be treated with respect in ways considered to be free of verbal abuse [6, 9] and disrespect of their opinions and voices [10]; right to be actively engaged with decisions on medical care and recruitment into medical research, experimental procedures or clinical trials [10]; and the right to quality care, which is a complex consort of variables that are often not easy to measure [11].

Clients receiving services in donor funded health care centres in Nigeria seem to experience less violation of their rights [12]. Donors like the U.S. President’s Emergency Plan for AIDS Relief (PEPFAR) and the Global Fund to fight AIDS, Tuberculosis and Malaria among others, have invested significantly to improve the quality of HIV service delivery in Nigeria using results-based performance models [13]. The use of the quality of care indicators to conduct annual assessment of compliance with the National antiretroviral guidelines helped improve the quality of HIV care delivered in the country [14]. The annual assessment was supported by the United States Centers for Disease Control and Prevention with the aim of promoting the delivery of appropriate care and treatment to individuals living with HIV through understanding of the human resource and infrastructure needs [14].

The huge donor investments in the delivery of HIV services in Nigeria should have significantly improved the quality of service delivery [15]. This has not been the case. The delivery of health care services through a public-health based approach rather than a human-rights based approach may have, inadvertently undermined the impact of the HIV care program in Nigeria and may have contributed to the limited success observed [16].

The risk of violating the rights of a significant number of people in need of HIV prevention and treatment services and sexual and reproductive health (SRH) services in Nigeria is likely to be high. Nigeria has an estimated HIV prevalence of 1.3%, translating to an estimated 1.9 million people living with HIV [17]. The HIV incidence rate was 0.42 per 1,000 population for all ages, and 0.06 per 1,000 for the adult population 15–49 years in 2021 [18]. Singles, female sex workers and men who have sex with men constitute a large proportion of the clients in need of HIV and SRH services because of the disproportionate risk for sexually transmitted infections and HIV they experience [19, 20]; they account for about 91% of all new infections among the adult population [18]. Also, key populations - female sex workers, men who have sex with men and people who inject drugs - account for about 11% of new HIV infections although they represent less than 2% of the total population [21].

These prevalence populations translate to large number of people at risk for HIV infection. UNAIDS estimates that there are 29,470 men who have sex with men (gay, bisexual, and other cisgender men) in Nigeria though Facebook’s estimate is 2.8 million men (> 18 years of age) interested in men or men and women and 180,000 men (> 18 years of age) interested in men in Nigeria in 2018 [22]. In 2014, there were an estimated 126,489 female sex workers in seven of the 36 states in Nigeria [23]. In 2018, the number of people who inject drugs in Nigeria was 80,000 [24]. There are no reliable estimates of transgender individuals in the country [25]. These populations who are often at high risk for HIV infection – singles (who are often adolescents and young persons, sexual (gay, bisexual, queer) and gender minority (transgender) individuals [26] – are often stigmatised by the society in Nigeria [27–32].

There is little known about clients’ perspectives on the violation of their rights to access quality HIV and SRH services in Nigeria. The human rights lens provides a means to examine systemic issues and state responsibility; and enables attention to be paid to the rights of socially excluded groups to receive discrimination-free health care. To optimize the health of citizens, and to learn how best to optimize facilitators and minimize barriers to access to care, we need to learn more from the clients’ perspectives.

This assessment was based on the 11 rights articulated in the “patients’ rights” charters developed for patients by PEPFAR in Nigeria. The rights were clustered into two groups – the rights to autonomy (five rights) and the rights to access care-related services (six rights). The aim was to determine the proportion of respondents whose experienced the violation of their rights to autonomy whilst accessing HIV and SRH services in clinics in Nigeria. The right to autonomy infers that patients have the ability to make independent decisions and choices about their health care [33]. Specifically, the study determined the types of rights to autonomy violated, and the profiles of those with a likelihood of experiencing the violation of their rights to autonomy.

## Methods

### Ethical consideration

Approval for the conduct of this study was obtained from the Institute of Public Health Obafemi Awolowo University Research Ethics Committee (IPHOAU/12/1606). The survey was preceded by an introduction about the study team, study objectives, and time needed to complete the questionnaire. This was followed by a consent form assuring participants of the confidentiality of their responses and emphasizing that their participation was voluntary. Only participants who consented to study participation by ticking a checkbox could proceed to the survey. The survey instrument was self- administered and filled anonymously online. The questions were close-ended. The study provided a waiver of parental consent for this non-invasive online HIV and sexual and reproductive health study in line with national ethics regulation that notes: *To participate in non-therapeutic research, persons aged 9 years and under require only parental consent while persons aged 10–12, require parental consent as well as assent from the YP. However, persons aged 13 and above and emancipated minors can consent for themselves without parental consent* [34].

### Study design and study population

The study was designed by the Coalition of Civil Society Networks on HIV and AIDS in Nigeria, a body of HIV community service organisations in Nigeria. The data was generated through an online survey (Survey Monkey®) with the aim of seeking the perspectives of respondents on the ease of access and quality of HIV prevention, treatment and ancillary care services, respect for rights, payment for services, and stigma. The survey was conducted in the nine States in Nigeria with highest prevalence of HIV in 2019 namely: Akwa Ibom, Anambra, Benue, Delta, Imo, Kaduna, Lagos, Rivers and Taraba States.

### Sample size calculation

The sample size was calculated based on the prevalence of 37.2% of general population reporting a violation of their rights to respect [35], using a desired precision of estimate of 0.05 with a confidence level of 95% for an infinite population size [36]. The pre-survey minimum sample size for this study was set at 250 valid respondents from each of the nine States, corresponding to a minimum sample size of 2250 participants. The study took consideration of the realities on the ground at the time of the survey such as resurgence of the COVID-19 pandemic due to the Delta variant, the containment measures such as transport limitation and took cognisance of the possibility for missing responses in the absence of guidance, support and motivation for survey response and increased the sample size by 10% to 2,475 [37]. From the statistical modelling perspective, we tried to have a minimum of 10 participants with complete responses per each of the 10 dependent variables for the study enabling us to perform regression analyses with a minimum probability level (p-value) of 0.05 [38].

### Study participants’ recruitment

The details of the study participants’ recruitment procedure had earlier been described by Folayan et al. [39]. Community representatives were drawn from the community of women, adolescents and young people, and key populations (female sex workers, transgender individuals, injecting drug users and men who have sex with other men) and members of the general population irrespective of their HIV status. Five community representatives were engaged to recruit eligible community members for the study in each of the nine states. Recruitment was through exponential non-discriminatory snowballing [40], and through crowdsourcing by placing calls through to community members and making contacting using social media platforms like Facebook, WhatsApp and email to reach eligible participants. Community representatives for female sex workers, transgender individuals, injecting drug users and men who had sex with other men for each of the nine states were identified by the National Umbrella Network for the target populations for the study. Community representative for the general populations, including adolescents and young people, were identified by the network of Civil Societies working on HIV and AIDS in Nigeria. The networks have secretariats in the nine target states. The community leaders selected as community representatives had large and diverse contacts in the state in view of their leadership positions.

The representatives were trained on the study protocol, ethical considerations for conducting online surveys, and effective communication. The trained community representatives worked with community members to discuss the study and promote the online study participation. The online survey was launched on February 7, 2021 and remained open until February 19, 2021. The study recruited respondents who were members of support groups, and service recipients in health care delivery service points in the target states. Respondents had to be able to read English, have access to the internet, and consented to participate in the study to be eligible to take the survey.

Trained community representatives, introduced to the study to eligible community members and shared their link to the online survey with them or posted their links on social media platforms. Peers were also encouraged to share the link with other peers in the State. The online study recruitment process was used in order to comply with the social distancing directive in place during the COVID-19 pandemic.

Restrictions were applied to IP settings of electronic devices used for the survey so that each participant could take the survey only once using the device. Participants could edit their responses freely until they chose to submit. Email addresses were not collected to ensure anonymity.

### Survey questionnaire

The questionnaire for the survey was developed by pooling together standardised tools used for assessing satisfaction with health care service delivery. The tool was reviewed first by two experts with a history of working with civil society organisations. The revised tool was then reviewed by the five-person steering committee constituted by the Coalition of Civil Society Networks on HIV and AIDS in Nigeria for this project. The steering committee consisted of the national representatives of the community of people living with HIV, women living with HIV, key populations, general populations and a representative from UNAIDS. The questionnaire was then reviewed by the 45 community representatives trained to collect data on the field. Some words were revised for context appropriateness. Finally, each of the 45 community representatives administered the tool to two persons in their community to identify the length of time spent for administering the questionnaire, and to identify if there was any other wordsmithing or question order sequencing required for the finalisation of the study questionnaire.

The finalised questionnaire had 47 questions divided into six sections. These sections included one that collected data on the sociodemographic variables (seven questions) and another that asked questions on the violation of patients’ rights to autonomy (five questions) and access to services (six questions). For this study, only data on the sociodemographic variables and the rights to autonomy were analysed. The findings from the data generated from the other four sections (ease of access to HIV prevention services; ease of access to ancillary care services; ease of access to HIV treatment services, satisfaction with HIV prevention; ancillary care services and HIV treatment services) are to be presented in other publications. The questions on the violation of patients’ rights to autonomy and access to services were contents of the “patients’ rights” charters developed by PEPFAR for routine use and training for service recipients at HIV prevention service points in Nigeria. The training was conducted by the Network of People Living with HIV and AIDS in Nigeria as part of the curriculum for training its support group members in Nigeria; and by providers of services to key populations.

### Dependent variables

#### Patients’ rights to autonomy

Information on the violation of rights to autonomy was assessed by the following questions: Has any of these your rights been violated during receipt of HIV prevention and treatment services in any facility in Nigeria? (a) Right to privacy and confidentiality of medical records; (b) Right to be treated with respect, regardless of gender, race, religion, ethnicity, allegations of crime, disability or economic circumstances; (c) Right to decline or consent to participation in medical research, experimental procedures or clinical trials; (d) Right to quality care in accordance to prevailing standards; (e) Right to complain and express dissatisfaction regarding services received. All these questions had responses “yes”, “no” and “don’t know”. For this analysis, only participants whose responses were “yes” or “no” were extracted.

### Independent variables

#### Sexual identity

Information on sexual identity of respondents (heterosexual/straight, gay, lesbian, bisexual, queer, prefer not to say) were also extracted. Respondents were asked to identify their sexual identity by ticking a checkbox. For this study, individuals who identified as being gay, lesbian, bisexual, or queer were categorized as a “sexual minority”.

#### HIV status

This was assessed by a single question on self-reported HIV status with responses including opting to identify as: “positive”, “negative”, “do not know” and “prefer not to report”. For the logistic regression analysis, data from respondents who preferred not to report their HIV status were excluded from the regression analysis. Respondents who reported that they did not know their HIV status were treated as a distinct HIV status entity because a prior study had demonstrated that men with unknown HIV status have a distinct profile from men who are HIV-negative or HIV-positive [41].

#### Living with disability

Respondents were asked to identify if they were living with a disability or not by checking a box (yes/no).

#### Access points to HIV services

Respondents were asked who funds the operations of the facilities they attended for HIV and SRH services by checking a box with the following options: Nigerian government facility, private facility, faith-based organization, PEPFAR, Global Fund, and “don’t know”. For the analysis, the responses were dichotomized into public (Nigerian government) and donor funded/private (all other options) facilities.

### Confounders

#### Sociodemographic variables

Data on age at last birthday (in years), education (no formal education, primary, secondary, tertiary), sex at birth (male, female, intersex, no response), marital status (single, married, separated/divorced or cohabiting) and gender identity (male, female, transgender) were collected. Respondents checked a box to indicate their sociodemographic profile.

#### Data analysis

Descriptive statistics were calculated as means and standard deviations or as frequencies and percentages. Tests of associations were conducted between the dependent (respect for patients’ right to autonomy), independent, and confounding variables. Five multivariate regression models were developed: one for each right to autonomy. The models were adjusted for age, sex assigned at birth, marital status and education level. Odd ratios/regression coefficients and their 95% confidence intervals (CI) were calculated. The IBM Statistical Package for Social Sciences, software version 23 was used for statistical analysis. Significance was set at < 5%.

## Results

There were 3197 individuals who accessed the survey of which 2451 (76.7%) respondents completed the survey and 2119 (86.5%) responded to the variables of interest for this study. The age of the respondents ranged from 13 years to 72 years with a mean and standard deviation of 30.10 and 8.14 years respectively. The sample included 1272 (60.0%) sexual minority individuals, 117 (5.5%) transgender individuals, 1413 (66.7%) individuals living with HIV, 104 (4.9%) individuals with disability and 940 (44.4%) individuals who accessed HIV services in donor funded/private facilities.

A total of 784 (34.9%) participants experienced violation of their right to be treated with privacy and confidentiality of medical records, 831 (36.8%) experienced violation of their right to be treated with respect, 742 (23.2%) experienced violation of their right to quality care in accordance to prevailing standards, 835 (37.4%) experienced violation of their right to complain and express dissatisfaction regarding services received and 690 (21.6%) experienced violation of their right to decline to consent to take part in medical research, experimental procedures or clinical trials.

### Right to privacy and confidentiality of medical records

As shown in Table 1, transgender individuals had significantly higher odds of experiencing violation of their right to privacy and confidentiality of medical records compared to individuals who were males (AOR: 1.70; 95% CI:1.12–2.57; p = 0.01). Also, older participants had a small but statistically significant higher odds of experiencing rights violation (AOR: 1.01; 95%CI: 1.00-1.03; p = 0.01). Respondents who were married (AOR:0.69; 95% CI: 0.55-087; p < 0.001), separated/divorced (AOR: 0.45; 95%CI: 0.29–0.71; p < 0.001), or cohabiting (AOR: 0.36; 95%CI: 0.21–0.62; p < 0.001) had significantly lower odds of experiencing violation of their right to privacy and confidentiality of medical records compared to individuals who were single.

### Right to be treated with respect

Females had significantly higher odds of experiencing violation of their right to be treated with respect compared to males (AOR: 1.42; 95% CI: 1.04–1.95; p = 0.03). Also, transgender individuals had significantly higher odds of experiencing violation of their right to be treated with respect compared to males (AOR:1.71; 95% CI: 1.13–2.59; p < 0.01). Respondents who were married (AOR: 0.76; 95%CI: 0.61–0.96; p = 0.02), separated/divorced (AOR: 0.62; 95%CI: 0.41–0.94; p = 0.02) and cohabiting (AOR: 0.35; 95% CI: 0.21–0.61; p < 0.001) had significantly lower odds of experiencing violation of their right to be treated with respect compared to singles.

### Right to quality care in accordance to prevailing standards

Respondents who were married (AOR: 0.74; 95% CI: 0.58–0.93; p = 0.01), separated/divorced (AOR: 0.58; 95%CI: 0.38–0.90; p = 0.02), cohabiting (AOR: 0.25; 95% CI: 0.13–0.47; p < 0.001) had significantly lower odds of experiencing violation of right to quality care in accordance to prevailing standards compared to singles. Also, participants who had secondary education (AOR: 0.54; 95%CI: 0.32–0.92; p = 0.02) had significantly lower odds of experiencing violation of their right to quality care in accordance to prevailing standards compared to those who had no formal education.

### Right to complain and express dissatisfaction regarding services received

Females had significantly higher odds of experiencing violation experiencing their right to complain and express dissatisfaction regarding services received compared to males (AOR: 1.39; 95% CI: 1.01–1.90; p = 0.04). Also, transgender individuals (AOR:1.57; 95% CI: 1.04–2.38; p = 0.03) had significantly higher odds of experiencing their violation of their right to complain and express dissatisfaction regarding services received violated compared to males. Respondents who were married (AOR: 0.76: 95%CI: 0.60–0.96; p = 0.02), separated/divorced (AOR: 0.53; 95%CI: 0.35–0.82; p < 0.001), cohabiting (AOR:0.28; 95%CI: 0.16–0.50; p < 0.001) had significantly lower odds of experiencing the violation of their right to complain and express dissatisfaction regarding services received compared to singles.

### Right to decline consent to participation in medical research, experimental procedures or clinical trials

Transgender individuals (AOR:1.81; 95%CI: 1.19–2.75; p < 0.001) had higher odds of experiencing violation of their right to decline to consent to participate in medical research, experimental research, experimental procedures or clinical trials compared to males. Respondents who were married (AOR: 0.73; 95%CI: 0.57–0.93; p = 0.01), separated/divorced (AOR: 0.55; 95%CI: 0.35–0.86; p < 0.001), cohabiting (AOR:0.24; 95% CI:0.13–0.47; p < 0.001) had lower odds of experiencing violation of their right to decline consent to participation in medical research, experimental procedures or clinical trials compared to those who were single.

**Table 1:** Binary logistic regression analysis of the factors associated with the violation of rights to care in Nigeria (N = 2119)

Variables	Total 2119 n(%)	Violation of right to privacy and confidentiality of medical records		AOR: 95% CI; p-value	Violation of right to be treated with respect		AOR: 95% CI; p-value
		Yes N = 718 n (%)	No N = 1332 n (%)		Yes N = 797 n (%)	No N = 1322 n (%)	
Age	2119 (100)	29.94 ± 7.92	30.25 ± 8.30	1.02 (1.00-1.03); 0.013	29.97 ± 7.78	30.24 ± 8.39	1.01 (0.99–1.02); 0.15
**Sex at birth**							
Male	796 (38.8)	305 (38.3)	491 (61.7)	1	314 (39.4)	482 (60.6)	1
Female	939 (45.8)	319 (34.0)	620 (66.0)	1.29 (0.93–1.77); 0.13	350 (37.3)	589 (62.7)	1.42 (1.04–1.95); 0.03
Intersex	315 (15.4)	94 (29.8)	221 (70.2)	1.37 (0.96–1.95); 0.09	98 (31.1)	217 (68.9)	1.20 (0.85–1.71); 0.30
**Educational status**							
No formal education	62 (2.9)	28 (45.2)	34 (54.8)	1	26 (41.9)	36 (58.1)	1
Primary	104 (4.9)	38 (36.5)	66 (63.5)	0.75 (0.39–1.43); 0.38	42 (40.4)	62 (59.6)	1.04 (0.55–1.99); 0.90
Secondary	786 (37.1)	263 (33.5)	523 (66.5)	0.64 (0.38–1.09); 0.10	266 (33.8)	520 (66.2)	0.76 (0.45–1.30); 0.31
Tertiary	1167 (55.1)	425 (36.4)	742 (63.6)	0.68 (0.40–1.15); 0.15	463 (39.7)	704 (60.3)	0.93 (0.55–1.58); 0.80
**Marital Status**							
Single	1173 (55.4)	456 (38.9)	717 (61.1)	1	473 (40.3)	700 (59.7)	1
Married	731 (34.5)	246 (33.7)	485 (66.3)	0.71 (0.57–0.90); 0.005	263 (36.0)	468 (64.0)	0.78 (0.62–0.99); 0.04
Separated/Divorced	129 (6.1)	34 (26.4)	95 (73.6)	0.47 (0.30–0.73); 0.01	42 (32.6)	87 (67.4)	0.64 (0.42–0.97); 0.04
Cohabiting	86 (4.1)	18 (20.9)	68 (79.1)	0.37 (0.21–0.63); <0.001)	19 (22.1)	67 (77.9)	0.36 (0.21–0.62(; <0.001)
**Gender Identity**							
Man	843 (39.8)	304 (36.1)	539 (63.9)	1	319 (37.8)	524 (62.2)	1
Woman	1159 (54.7)	394 (34.0)	765 (66.0)	1.07 (0.76–1.50); 0.70	418 (36.1)	741 (63.9)	0.90 (0.65–1.26); 0.54
Transgender individual	117 (5.5)	56 (47.9)	61 (52.1)	1.70 (1.13–2.56); 0.01	60 (51.3)	57 (48.7)	1.74 (1.16–2.63); <0.001
**Sexual orientation**							
Heterosexuals	717 (46.9)	243 (33.9)	474 (66.1)	1	259 (36.1)	458 (63.9)	1
Sexual minorities	813 (55.2)	300 (36.9)	513 (63.1)	1.05 (0.86–1.29); 0.65	317 (39.0)	496 (61.0)	1.05 (0.86–1.28); 0.66
**Access to HIV services**							
Public	1179 (55.6)	419 (35.5)	760 (64.5)	1	455 (38.6)	724 (61.4)	1
Donor funded/Private	940 (44.4)	335 (35.6)	605 (64.4)	0.99 (0.82–1.19); 0.91	342 (36.4)	598 (63.6)	0.88 (0.73–1.06); 0.17
**Living with disability**							
No	2015 (95.1)	714 (35.4)	1301 (64.6)	1	762 (37.8)	1253 (62.2)	1
Yes	104 (4.9)	40 (38.5)	64 (61.5)	1.12 (0.74–1.7); 0.59	35 (33.7)	69 (66.3)	0.81 (0.53–1.24); 0.32
**HIV**							
Negative	669 (31.6)	234 (31.0)	435 (65.5)	1	254 (38.0)	415 (62.0)	1
Positive	1413 (66.7)	505 (35.7)	908 (64.3)	1.10 (0.90–1.36); 0.36	528 (37.4)	885 (62.6)	1.03 (0.84–1.27); 0.78
Don’t know	37 (1.7)	15 (40.5)	22 (59.5)	1.22 (0.74–1.7); 0.57	15 (40.5)	22 (59.5)	1.15 (0.58–2.28); 0.70

**Table:** (continued): Binary logistic regression analysis of the factors associated with the violation of rights to care in Nigeria (N=2119)

Variables	Total 2119 n(%)	Violation of right to quality care in accordance to prevailing standards		AOR: 95% CI; p-value	Violation of right to complain and express dissatisfaction and regarding services received		AOR: 95% CI; p-value	Violation of right to decline consent to participation in medical research, experimental procedures or clinical trials		AOR: 95% CI; p-value
		Yes N = 666 n (%)	No N = 1384 n (%)		Yes N = 778 n (%)	No N = 1341 n (%)		Yes N = 627 n (%)	No N = 1423 n (%)	
Age	2119 (100)	30.00 ± 7.80	30.21 ± 8.35	1.01 (0.99–1.02); 0.12	29.89 ± 7.82	30.28 ± 8.36	1.01 (0.99–1.02); 0.15	30.04 ± 7.75	30.18 ± 8.35	1.01 (1.00-1.03);0.04
**Sex at birth**										
Male	796 (38.8)	266 (33.4)	530 (66.6)	1	305 (38.3)	491 (61.7)	1	256 (32.2)	540 (67.8)	1
Female	939 (45.8)		636 (67.7)	1.26 (0.91–1.74); 0.16		601 (64.0)	1.39 (1.01–1.90); 0.04	283 (30.1)	656 (69.9)	1.21 (0.87–1.69); 0.25
Intersex	315 (15.4)	303 (32.2) 97 (30.8)	218 (69.2)	0.99 (0.69–1.41); 0.95	338 (36.0) 99 (31.4)	216 (68.6)	1.32 (0.92–1.87); 0.13	88 (27.9)	227 (72.1)	1.04 (0.72–1.50); 0.83
**Educational status**										
No formal education	62 (2.9)	28 (45.2)	34 (54.8)	1	27 (43.5)	35 (56.5)	1	23 (37.1)	39 (62.9)	1
Primary	104 (4.9)	39 (37.5)	65 (62.5)	0.79 (0.42–1.51); 0.48	41 (39.4)	63 (60.6)	0.92 (0.48–1.76); 0.80	35 (33.7)	69 (66.3)	0.93 (0.48–1.81); 0.75
Secondary	786 (37.1)	236 (30.0)	550 (70.0)	0.54 (0.32–0.92); 0.02	272 (34.6)	514 (65.4)	0.72 (0.43–1.23); 0.23	234 (29.8)	552 (70.2)	0.75 (0.44–1.30); 0.31
Tertiary	1167 (55.1)	397 (34.0)	770 (66.0)	0.63 (0.37–1.06); 0.08	438 (37.5)	729 (62.5)	0.78 (0.46–1.32); 0.36	361 (30.9)	806 (69.1)	0.76 (0.45–1.31); 0.77
**Marital Status**										
Single	1173 (55.4)	420 (35.8)	753 (64.2)	1	393 (33.5)	780 (66.5)	1	393 (33.5)	780 (66.5)	1
Married	731 (34.5)	231 (31.6)	500 (68.4)	0.75 (0.59–0.95); 0.02	216 (29.5)	515 (70.5)	0.78 (0.62–0.99); 0.04	216 (29.5)	515 (70.5)	0.74 (0.58–0.94); 0.01
Separated/Divorced	129 (6.1)	37 (28.7)	92 (71.3)	0.59 (0.39–0.92); 0.02	33 (25.6)	96 (74.4)	0.56 (0.36–0.85); <0.001	33 (25.6)	96 (74.4)	0.56 (0.36–0.87); 0.01
Cohabiting	86 (4.1)	12 (14.0)	74 (86.0)	0.25 (0.14–0.48); <0.001	11 (12.8)	75 (87.2)	0.29 (0.16–0.51) < 0.001	11 (12.8)	75 (87.2)	0.25 (0.13–0.48); <0.001
**Gender Identity**						61				
Man	843 (39.8)	279 (33.1)	564 (66.9)	1	311 (36.9)	532 (63.1)	1	260 (30.8)	583 (69.2)	1
Woman	1159 (54.7)	372 (32.1)	787 (67.9)	0.88 (0.62–1.23); 0.44	411 (35.5)	748 (64.5)	1.00 (0.71–1.39); 0.99	341 (29.4)	818 (70.6)	0.89 (0.63–1.26); 0.52
Transgender individual	117 (5.5)	49 (41.9)	68 (58.1)	1.44 (0.95–2.18); 0.09	56 (47.9)	62 (52.1)	1.61 (1.07–2.44); 0.02	52 (44.4)	65 (55.6)	1.84 (1.21–2.79); <0.001
**Sexual identify**										
Heterosexuals	717 (46.9)	212 (29.6)	505 (70.4)	1	248 (34.6)	469 (65.4)	1	205 (28.6)	512 (28.6)	1
Sexual minorities	813 (53.1)	273 (33.6)	540 (66.4)	1.14 (0.92–1.40); 0.23	312 (38.4)	501 (61.6)	1.14 (0.93–1.39); 0.22	261 (32.1)	552 (67.9)	1.05 (0.85–1.30); 0.66
**Access to HIV services**										
Public	1179 (55.6)	401 (34.0)	778 (66.0)	1	454 (38.5)	725 (61.5)	1	359 (30.4)	820 (69.6)	1
Donor funded /Private	940 (44.4)	299 (31.8)	641 (68.2)	0.88 (0.73–1.07); 0.21	324 (34.5)	616 (65.5)	0.82 (0.68–0.98); 0.03	294 (31.3)	646 (68.7)	1.05 (0.86–1.27); 0.64
**Living with disability**										
No	2015 (95.1)	668 (33.2)	1347 (66.8)	1	739 (36.7)	1276(63.3)	1	623 (30.9)	1392 (69.1)	1
Yes	104 (4.9)	32 (30.8)	72 (69.2)	0.86 (0.56–1.33); 0.50	39 (37.5)	65 (62.5)	0.99 (0.65–1.50); 0.95	30 (28.8)	74 (71.2)	0.89 (0.57–1.39); 0.60
**HIV**										
Negative	669 (31.6)	222 (33.2)	447 (66.8)	1	242 (36.2)	427 (63.8)	1	198 (29.6)	471 (70.4)	1
Positive	1413 (66.7)	467 (33.1)	946 (66.9)	1.04 (0.84–1.28); 0.74	523 (37.0)	890 (63.0)	1.05 (0.86–1.30); 0.63	445 (31.5)	968 (68.5)	1.16 (0.93–1.44); 0.19
Don’t know	37 (1.7)	11 (29.7)	26 (70.3)	0.88 (0.42–1.83) 0.73	13 (35.1)	24 (64.9)	0.94 (0.47–1.91); 0.07	10 (27.0)	27 (70.4)	0.87 (0.41–1.86); 0.73

## Discussion

The findings of this study indicates that over a third of respondents had their rights to autonomy violated when receiving HIV and SRH services in Nigeria. Gender identity and marital status were factors significantly associated with the violation of rights. Specifically, transgender individuals were more likely to have all their rights to autonomy violated when compared men. Also, those who were married, separated/divorced or cohabiting were less likely to all their rights violated when compared to singles. In addition, respondents who were females were more likely to have their rights to complain and express dissatisfaction regarding services received, and their right to be treated with respect violated when compared w males. Sexual identity, living with disability, HIV status and point of access to services were not significantly associated with the violation of rights.

These findings draw attention to the poor management of rights of HIV and SRH service users in Nigeria. The design and implementation of the primary study was led by community members representing different civil society organisations, including organisations working with people living with HIV, people with disability and key populations. The findings reflect an area of genuine concern and a likelihood that perspectives on the quality of service were authentic. The large sample size allowed for robust analysis, and the large database allowed for potential confounding factors to be accounted for within the statistical design. The questionnaire was pre-tested for clarity and acceptability, and the questions used for the assessment of patients’ rights were adopted from the patients’ right charter that clients accessing services in HIV clinics had been introduced too community members three years ahead of the study. These efforts reduced the risk for bias reporting.

Despite these strengths of the study, the response to the questionnaire was, however, restricted to English speakers with access to the internet and smartphone or similar device. This has implications for the generalisability of the study as this may have inevitably reduced access of those with lower educational attainment and socioeconomic status who are more vulnerable and more likely to experience violations to respond to the survey. The results reported here may therefore, under-represent the real magnitude of the problem. A non-probability sampling method that would reach vulnerable and hidden populations was used [42], which implies the study findings may be generalisable to a sub-set of the study population who are likely to have higher socioeconomic status, as reflected by the educational status of the respondents. In addition, the study did not categorise the data on transgender individuals as transgender men or transgender women, further limiting the scope of application of the study findings. Also, this was a cross-sectional study, so no causal inferences could be established. Despite these potential shortfalls inherent within the study design, the robust analysis makes the findings valuable for policy and programmatic considerations in Nigeria. It also helps to develop hypotheses that can be explored in further studies.

First, we identified that about a third of HIV and SRH service recipients reported a violation of their human rights. These violations did not differ significantly by sexual identity, disability, or by HIV positivity status. This finding contradicts prior projected population-specific discrimination of service recipients by these statuses. People living with HIV [43], people with disability [44] and individuals from sexual minority groups [45] have all reported that their rights have been violated when receiving health services. What the present study suggests is that rights violation is a common practice at health care service delivery points—both public and private practices—with no significant difference between the service recipients in both types of facilities. This is in concordance with previous reports that found a high prevalence of rights abuses in both public and private facilities [46]. Prior studies have also highlighted poor quality of health care service delivery in Nigeria due to experiences of rights violations [47]. The high prevalence of reports of rights violation at healthcare service delivery points may be a deterrent for preventive care service access in Nigeria, and a reason for delayed hospital management of multiple pathologies [48] contributing to the high prevalence of preventable deaths.

These study findings once again reinforce the need for the Government of Nigeria to reform the health care system using a rights-based approach [49]. This becomes even more critical with the inequality lens and people-centred approach proffered by the Global AIDS Strategy 2021–2026, as a strategy for ending AIDS [50]. The findings are also important because human rights considerations in patient care carry legal implications and can be demanded through judicial action. They also provide a powerful language to articulate and mobilize around justice concerns, and to engage in advocacy through media and political negotiation [2].

Community advocates can therefore use the evidence generated through this study to push for health sector reforms by making demands for client-friendly services using a human-right approach. Such health reforms should include hospital supervisory visits that monitor and report on the violation of clients’ rights; and the use of data generated from these supervisory visits to institute trainings and practice reforms that improves the respect of patients’ rights [51]. Respects for rights should include the recognition of transgender as a gender identity and intersex as a third birth sex. Nigeria had actively driven changes in health care practices to improve public and private sector care for key populations with some measure of success [52]. These drive for change needs to be more encompassing to address the needs of all minorities who may be at risk of receiving inequitable and discriminatory health services in the country. A collective community drive by health advocates for such reforms may be more impactful than an advocacy drive by populations worse affected by inequality because of concerns about the homophobic legal system in Nigeria if there may be the need for legal recourse to reform the health system [53].

Attention also needs to be paid to transgender individuals, who consistently reported higher likelihood of experiencing rights violations compared to men in this study. There is a small body of evidence about transgenderism in Nigeria [54–56] that suggest a high prevalence of HIV [54], sexually transmitted infections [57] as well as stigma and human rights violations, which hinders service utilization [28, 57]. This study provides the first quantitative report on the perspectives of transgender individuals on the violation of their rights to autonomy within the health care sector in Nigeria. Over 40% of transgender individuals reported violations of their rights in this study. Though we did not distinguish between perceived and/or enacted human rights violations, Sekoni et al. [28] provided evidence to indicate that transgender individuals do experience enacted rights violations that needs to be addressed. The needs of minority groups, including those of transgender individuals, need to be amplified to eliminate the risk of being left behind. This clarity is crucial for identifying the extent of necessity in developing distinct capacity building programs for health care providers to improve health care for transgender individuals; or if, indeed, programs should focus on addressing perceived stigma by transgender individuals.

The observed violation of the rights of females to complain and express dissatisfaction with services received and their right to be treated with respect is likely a reflection of the patriarchal Nigeria society where women have less rights to respect and are less able to voice a need for their rights [58]. This study contributes to a small but growing body of evidence on how patriarchal tendencies may affect the delivery of poor-quality services to women. In patriarchal societies, men often have power and control even within the health sector. The restrictive gender norms and gender inequalities are thereby, replicated and reinforced in health systems [59]. The result is that health systems reinforce patients’ traditional gender roles; and health care programmes are rarely gender responsive [59]. This may explain the poor satisfaction of women with the HIV and SRH care noted in this study. Health reform programs should also include gender responsive programming for clients with special attention paid to the peculiar needs of female patients.

Finally, being in a relationship appears to reduce the likelihood of experiencing rights violations within the sample population. This study again, contributes to existing literature that reports on the link between marital status and the quality of HIV and SRH care delivery. Married individuals have access to lower health care costs, increased access to resources that may affect service utilization such as health insurance and disposable income, engage in less risky health-related behaviours that may affect uptake of services, and have better health status and quality of life when compared to singles [60]. This is due to the concept of “spousal protection” emanating spouses function as care takers, providing physical and emotional support [61–63] and support more use of health care service [60]. Another factor may be that in the Nigerian culture, the society accords respect to married women [64] and this may translate to the respect of the rights of married individuals when receiving HIV and SRH services. Our study produces evidence suggesting that having a spousal relationship (past or present) can reduce the likelihood of experiencing the violation of rights when receiving HIV and SRH services, which may also contribute to prior observations on the “spousal protection” concept for health [65].

The study however, needs to be treated with caution. We recomputed the statistical power of our sample size based on the finding on the proportion of respondents whose rights were violated in this study having found no significant discrimination prevalence the violation of the rights of sexual minority individuals, people living with HIV and people with disability in public and private health institutions. We noted that the study has a power to detect a difference that ranged from 5.0 to 50.4% for the five rights violations studied by population and by type of service accessed. These powers suggest that that a larger sample size may be needed to detect a truly meaningful difference between the populations.

In conclusion, this study identified that the proportion of recipients of HIV and SRH services whose rights were violated was high within the sample population. This violation of rights do not significantly differ by sexual identity, HIV status, or disability status, but it differs by both gender identity and marital status. Transgender individuals had a higher likelihood of experiencing rights violation, and those in spousal relationships have a lower likelihood of experiencing rights violation. Future studies are need to identify how these risk indicators may inform the design and implementation of HIV and SRH services that are rights-respectful.

## Data Availability

All data generated for this study are presented in the manuscript. The dataset for the online study data can however be accessible on reasonable request from one the study author, Morenike Oluwatoyin Folayan, toyinukpong@yahoo.co.uk.

## Abbreviations

HIV: Human Immunodeficiency Syndrome.
SRH: Sexual and Reproductive Health.
PEPFAR: President’s Emergency Plan for AIDS Relief.
GFATM: Global Fund to Fight AIDS, Tuberculosis and Malaria.
WHO: World Health Organization.
HIVQUAL-N: Quality of HIV care- Nigeria.
AIDS: Acquired Immunodeficiency Syndrome.
CDC: Centers for Disease Control and Prevention.
UNAIDS: Joint United Nations Programme on HIV/AIDS.
CI: Confidence Interval.
SD: Standard Deviation.
AOR: Adjusted Odds Ratio.
